# Short-Term Dairy Product Elimination and Reintroduction Minimally Perturbs the Gut Microbiota in Self-Reported Lactose-Intolerant Adults

**DOI:** 10.1128/mbio.01051-22

**Published:** 2022-06-13

**Authors:** Courtney J. Smith, Les Dethlefsen, Christopher Gardner, Linda Nguyen, Marcus Feldman, Elizabeth K. Costello, Oren Kolodny, David A. Relman

**Affiliations:** a Department of Genetics, Stanford University School of Medicine, Stanford, California, USA; b Department of Medicine, Stanford University School of Medicine, Stanford, California, USA; c Department of Gastroenterology and Hepatology, Stanford University School of Medicine, Stanford, California, USA; d Department of Biology, Stanford University grid.471392.agrid.168010.egrid.471392.agrid.168010.e , Stanford, California, USA; e Department of Ecology, Evolution and Behavior, A. Silberman Institute of Life Sciences, Hebrew University of Jerusalem, Jerusalem, Israel; f Department of Microbiology & Immunology, Stanford University School of Medicine, Stanford, California, USA; g Infectious Diseases Section, Veterans Affairs Palo Alto Health Care System, Palo Alto, California, USA; University of California, Irvine

**Keywords:** diet, gut, lactose intolerance, microbial communities, microbiota

## Abstract

An outstanding question regarding the human gut microbiota is whether and how microbiota-directed interventions influence host phenotypic traits. Here, we employed a dietary intervention to probe this question in the context of lactose intolerance. To assess the effects of dietary dairy product elimination and (re)introduction on the microbiota and host phenotype, we studied 12 self-reported mildly lactose-intolerant adults with triweekly collection of fecal samples over a 12-week study period: 2 weeks of baseline diet, 4 weeks of dairy product elimination, and 6 weeks of gradual whole cow milk (re)introduction. Of the 12 subjects, 6 reported either no dairy or only lactose-free dairy product consumption. A clinical assay for lactose intolerance, the hydrogen breath test, was performed before and after each of these three study phases, and 16S rRNA gene amplicon sequencing was performed on all fecal samples. We found that none of the subjects showed change in a clinically defined measure of lactose tolerance. Similarly, fecal microbiota structure resisted modification. Although the mean fraction of the genus Bifidobacterium, a group known to metabolize lactose, increased slightly with milk (re)introduction (from 0.0125 to 0.0206; Wilcoxon *P = *0.068), the overall structure of each subject’s gut microbiota remained highly individualized and largely stable in the face of diet manipulation.

## INTRODUCTION

Lactose is a disaccharide and the most abundant carbohydrate found in mammalian milk. Lactase is an enzyme that allows utilization of lactose from milk by cleaving lactose into glucose and galactose since disaccharides are poorly absorbed. To utilize the lactose in mothers’ milk, mammals in infancy produce lactase at the brush border of the jejunum and then normally stop expressing this enzyme shortly after weaning. Undigested lactose passing through the small intestine into the colon can cause lactose intolerance, which manifests as diarrhea, abdominal discomfort, bloating, and flatulence following consumption of lactose-containing dairy products. These symptoms are likely the product of the osmotic load of undigested lactose in the colon, as well as gas and other metabolites from bacteria fermentation (1, 2).

In humans, genetic adaptations occurred in several populations approximately 4,000 to 10,000 years ago, following domestication of milk-producing ungulates, causing approximately 35% of the world’s population to continue expressing lactase into adulthood (3–8). So-called lactase persistence confers lactose tolerance, but tolerance can evidently be conferred by other mechanisms as well: many non-lactase-persistent individuals consume dairy products regularly without reporting any symptoms (1, 4, 9, 10). One potential mechanism of lactose breakdown in non-lactase-persistent individuals is by bacterial fermentation in the gut. In humans, genetic variation within the locus that includes the gene *LCT*, which encodes the enzyme lactase, has been shown to have an age-dependent, genome-wide significant association with abundance in the gut microbiota of the genus Bifidobacterium, a group known to metabolize lactose, even though host genetics has a minor role in determining microbiota structure relative to other environmental factors such as diet (11–13).

The lactose hydrogen breath test (HBT) is the primary diagnostic test for clinical lactose intolerance, wherein one measures the concentration of hydrogen and methane in the breath of patients for 3 h following the consumption of a standardized dose of lactose (typically 25 g, or the equivalent of about two cups of milk) (14, 15). The negative effects of dairy product consumption frequently cause intolerant individuals to avoid dairy products. Such avoidance has become a widespread recommendation for these individuals by medical practitioners and is growing in popularity among large segments of Western society. However, the regular consumption of dairy products (and the associated calcium intake) is linked to positive health outcomes, such as reduced risk of osteoporosis and bone fractures (16, 17). In addition, previous studies suggest that lactose tolerance can be acquired by lactose-intolerant individuals over a few weeks by the inclusion of regular or regularly increasing amounts of lactose in the diet (1, 18–23).

The importance of the role of the microbiota in health and disease is increasingly evident. Colorectal cancer, inflammatory bowel disease, and obesity are examples of conditions to which the gut microbiota contributes (18, 24). In the last decade, researchers have begun exploring this relationship more deeply through intervention studies designed to discover whether the structure of the gut microbiota can be manipulated in such a manner as to influence host phenotype (25, 26). Prior studies have suggested that lactose tolerance can be acquired via gradual introduction of dairy products into the diet, but little is known about the microbiota changes presumed to accompany and facilitate acquired tolerance (5, 19, 27, 28). Therefore, we examined changes in gut microbiota structure by manipulating dietary dairy product consumption. To date, changes in overall human gut microbiota structure in response to dietary changes regarding the consumption of dairy products have not been well studied.

Here, we combined gut microbiota surveys with HBT results to assess the responses of the microbiota to dietary manipulation of dairy product consumption. Our results demonstrate a surprising level of resistance to perturbation by dairy product interventions, in terms of both clinical lactose tolerance status and gut microbiota structure.

## RESULTS

### Overview of study design and sample collection.

**FIG 1:**
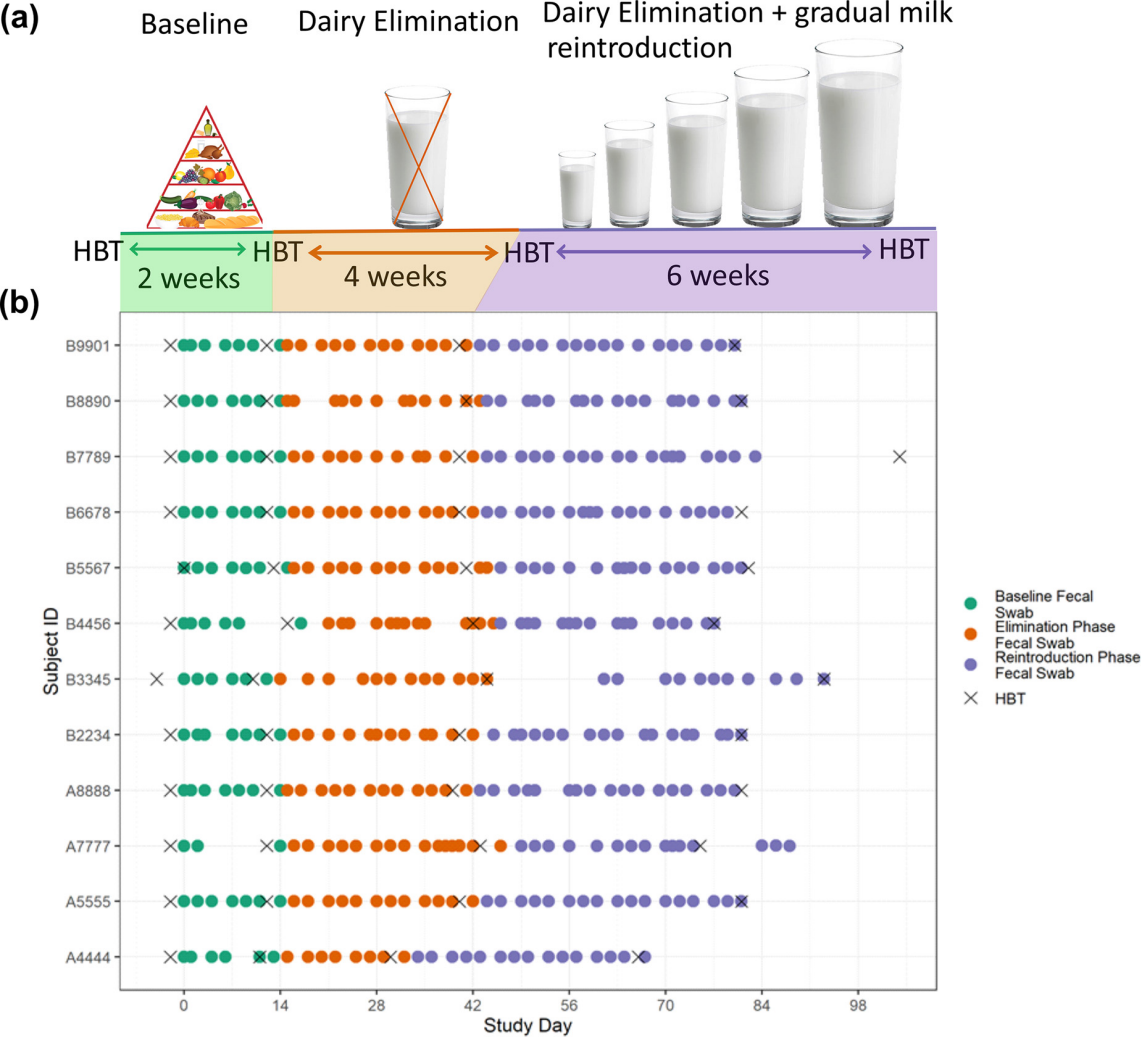
The study consisted of triweekly fecal sample collection and three diet phases with a hydrogen breath test (HBT) before and after each phase. (a) Overview of 12-week study design. (b) Sample collection (day 0 is the day the first fecal sample was collected). During the 2-week baseline phase, subjects maintained their normal diets. During the 4-week elimination phase, subjects continued their normal diets but avoided all dairy products. During the 6-week (re)introduction phase subjects were instructed to follow a specific protocol for gradually increasing consumption of whole cow milk, working up to two cups of milk a day during the last week.

We studied 12 self-reported mildly lactose-intolerant subjects (see Table S1 for subject information), each with triweekly collection of fecal samples over a 12-week study period (2 weeks of baseline diet, 4 weeks of complete dairy product elimination, and 6 weeks of gradual whole cow milk [re]introduction) and a HBT before and after each phase (Fig. 1). We recruited individuals who self-reported being lactose intolerant in order to enrich for non-lactase-persistent study subjects; 6 subjects, despite self-reporting as somewhat intolerant, regularly consumed some lactose-containing dairy products. The elimination phase served to ensure that lactose was absent in the participants’ diets regardless of prior intake and types of dairy products that had been consumed. All dairy products were excluded in this phase, even though some dairy products contain little to no lactose, to simplify and decrease unnecessary variation in the interpretation of the dietary instructions by the participants. Participants introduced or reintroduced specific, gradually increasing amounts of whole cow milk (standardized brand). This study design was chosen to ensure that during the (re)introduction phase, the change was coordinated and controlled across participants.

10.1128/mbio.01051-22.1 TABLE S1 Aggregate background information on subjects that participated in the study. Prior to the start of the study, A7777 and B4456 had no dairy products in their diet; B3345, B6678, B7789, and B8890 had only lactose-free dairy products; A4444, A5555, A8888, and B2234 had less than one serving per week of low-lactose-containing foods such as cheese, Greek yogurt, and butter (referred to as “some dairy”); and B5567 and B9901 regularly had lactose-containing dairy products. One lesson learned from this pilot study is that because the overall structure of each subject’s gut microbiota remained highly individualized and largely stable, a longer or more aggressive intervention may be needed to see a more obvious shift in microbiota structure for a study of this size. The aggressiveness of the whole cow milk (re)introduction intervention was limited by feasibility and ethical considerations because the subjects were self-identified lactose-intolerant human subjects and would understandably be deterred by too aggressive of a milk (re)introduction. Another lesson is that a longer baseline period allowing for additional baseline measurements of both microbiota structure and lactose intolerance symptoms is needed to better identify what constitutes normal baseline fluctuations in the absence of an intervention. This is especially important in the case of the HBT, in which the variation between the two baseline samples were often the same, if not more, than the variation from the postelimination and post(re)introduction samples. A possible explanation for this is that the HBT necessitated a major dietary restriction in the preceding 24-h period to each test and also required subjects to drink two cups of milk (see Materials and Methods). Therefore, because many of the subjects had not consumed significant amounts of dairy products in a long time, the first HBT at the beginning of the study might have been an unintentionally significant dietary intervention. A future way to identify whether this could have played a role in shifting the microbiota at the beginning of the study would be to start fecal swab sample collection prior to the first HBT. Download Table S1, XLSX file, 0.01 MB. Copyright © 2022 Smith et al. 2022 Smith et al. https://creativecommons.org/licenses/by/4.0/ This content is distributed under the terms of the Creative Commons Attribution 4.0 International license.

Throughout the 12 weeks of the study, the severity of lactose intolerance was evaluated with two metrics. The first was based on the HBT: we measured the combined concentration of hydrogen and methane gas in each breath sample collected for each subject over a 6-h period after drinking two cups of whole cow milk. This differed from the standard procedure for the HBT, which typically involves collection of breath samples for only 3 h after drinking a solution of lactose powder mixed with water. Each subject completed four HBTs at standard time points during the study, enabling temporal analysis within subject as well as between subjects. The second metric was based on self-reported symptoms recorded in a daily log throughout the study and every half hour during each HBT.

### Clinical status at baseline and in response to intervention.

At baseline, all subjects self-identified as mildly lactose intolerant, and 6 of 12 reported regularly consuming some amount of dairy products in their diet (although 4 of them had less than one serving per week), while the others abstained from dairy products completely or used lactose-free products. We hypothesized that subjects who regularly consume some dairy products in their diet might lose or see reductions in the relative abundances of lactose-utilizing bacteria during the dairy product elimination phase, thus becoming somewhat more lactose intolerant compared to baseline, and that subjects might increase lactose tolerance during the (re)introduction phase if the milk consumed during this phase was sufficient to increase the growth of specialized bacteria that metabolize lactose with few ill effects for their host.

**FIG 2:**
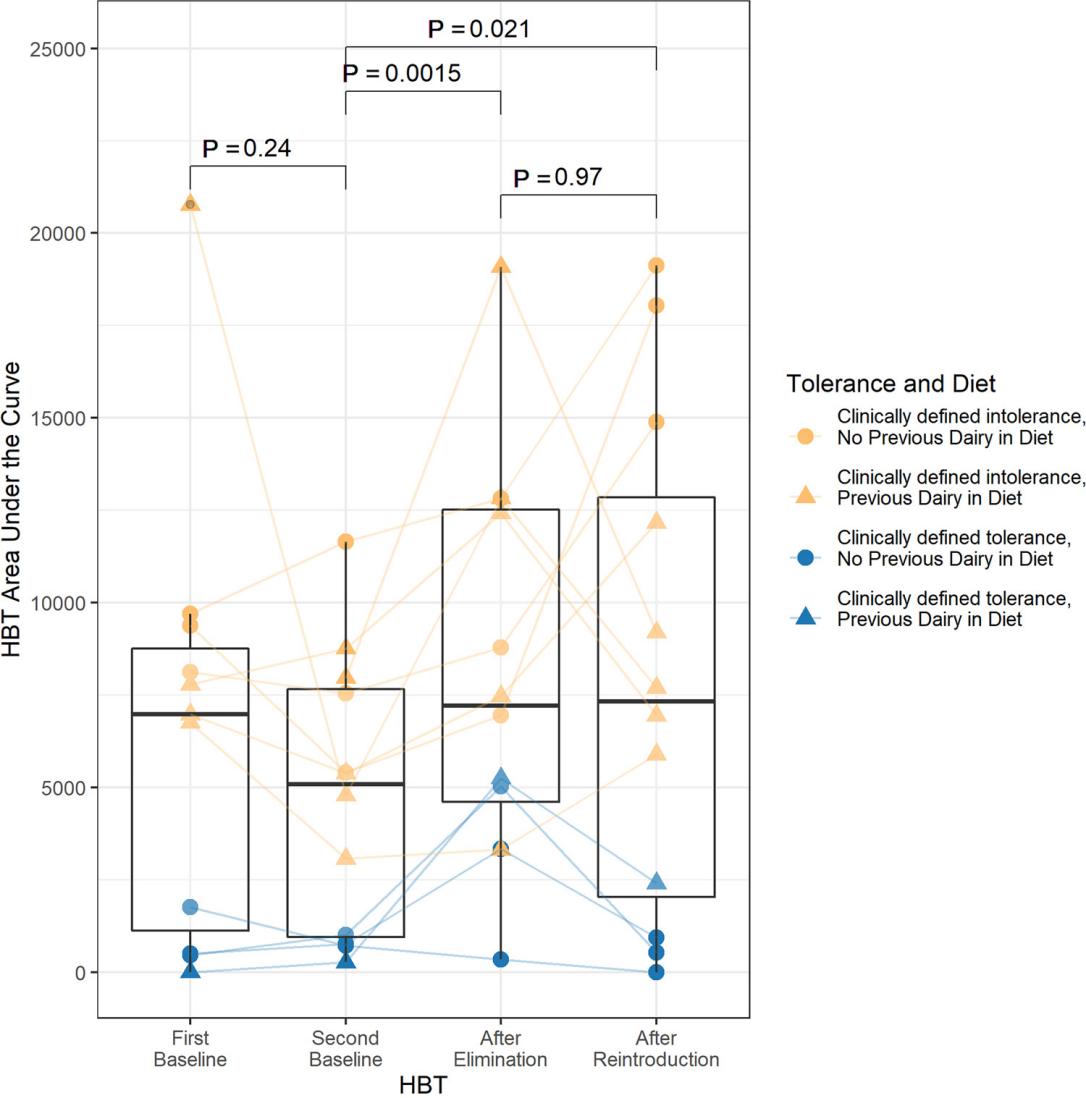
Area under the curve (AUC) of the hydrogen breath test (HBT) for each subject across the study. AUC significantly increased (relative to the second baseline) after the dairy product elimination phase, potentially reflecting an increase in intolerance, despite no change in clinical classification. After the (re)introduction phase, some increased further, while others seemed to recover. Coloring indicates the clinically defined intolerance status based on HBT results, and shape indicates whether subjects were dairy product abstainers (circles) or consumers (triangles) prior to the study. See Materials and Methods for details of HBT AUC calculation. Paired sample Wilcoxon signed-rank test *P* values are shown.

Each HBT (Fig. 1) included 13 breath samples taken at 30-min intervals over 6 h; thus, the combined concentration of hydrogen and methane gas of each sample could be plotted against time (see Fig. S1 for plot). Subjects were classified by the HBT as clinically intolerant of lactose if the concentrations at any of these time points were greater than 20 ppm above the concentration for that individual at time zero. At baseline, the HBT classified 8 of the 12 subjects as lactose intolerant, despite all 12 self-reporting lactose intolerance, and the clinical status did not change for any subject during the study. We developed a quantitative measure of lactose tolerance rather than relying on a dichotomous clinical classification, and so we calculated the change in area under the HBT curve (AUC) over time, as this reflects the total hydrogen and methane gas concentration over the 6-h collection period for each HBT. We observed a significant increase in AUC between the second baseline HBT and the HBT after the dairy product elimination phase (paired Wilcoxon signed-rank test; *P* = 0.0015) (Fig. 2; see Fig. S2 for raw results). Of the 12 subjects, 11 had a higher AUC in the HBT after the dairy product elimination phase than in the second baseline HBT, including all 8 subjects with clinically defined lactose intolerance (paired Wilcoxon signed-rank test; *P* = 0.008). We also observed a significant increase in AUC between the second baseline HBT and the HBT after the (re)introduction phase (paired Wilcoxon signed-rank test; *P* = 0.021). During the HBT after the (re)introduction phase, 9 of the 12 subjects had a higher AUC than during the second baseline HBT, including 7 of the 8 subjects with clinically defined lactose intolerance (paired Wilcoxon signed-rank test; *P* = 0.023).

10.1128/mbio.01051-22.3 FIG S1 Hydrogen breath test (HBT) results for each subject showing the combined concentration of hydrogen and methane gas in their breath (with the baseline sample value subtracted from each or set to 0 if adjusted value was negative) as time elapsed after drinking two cups of milk. Individuals are classified by the HBT as lactose intolerant in the clinic if the sum of hydrogen and methane concentrations in their breath sample reaches greater than 20 ppm above their first sample concentration at any time point during the HBT. At baseline, the HBT classified 8 of the 12 subjects as lactose intolerant, and none of the subjects changed their initial status of lactose tolerance during the study tests. HBT baseline results for subjects A7777 and B5567 are not included because the breath sample collection was not completed properly. Download FIG S1, TIF file, 1.7 MB. Copyright © 2022 Smith et al. 2022 Smith et al. https://creativecommons.org/licenses/by/4.0/ This content is distributed under the terms of the Creative Commons Attribution 4.0 International license.

10.1128/mbio.01051-22.4 FIG S2 (a) Individual results for area under the curve (AUC) of the hydrogen breath test (HBT) for each subject across the study. (b) Aggregate AUC of the HBT for each subject showing raw values instead of those shown in Fig. 2, in which any negative calculated breath sample (after subtracting the concentration measured in a given breath sample from the baseline sample concentration for that test) was set to 0 (see Materials and Methods for further information). As shown in Fig. 2, subjects’ HBT AUC significantly increased after the dairy product elimination phase, potentially reflecting an increase in intolerance. Download FIG S2, TIF file, 2.2 MB. Copyright © 2022 Smith et al. 2022 Smith et al. https://creativecommons.org/licenses/by/4.0/ This content is distributed under the terms of the Creative Commons Attribution 4.0 International license.

Interestingly, based on the self-reported symptom data, most subjects reached high qualitative self-reported lactose tolerance by the end of the milk (re)introduction period relative to the baseline period, revealing an unexpected discordance between clinical lactose tolerance as assessed by HBT and self-reported lactose tolerance defined by symptoms. In the final week of the study, 9 of 12 subjects (including 5 of the 8 subjects defined as clinically lactose intolerant by the HBT) were able to tolerate two cups of milk daily without any reported symptoms (except mild gassiness), and the symptoms reported by the others over this phase were not severe (see Table S2 for symptoms summary; see Fig. S3 for relation between symptoms and HBT AUC). However, the implications of these findings are limited in the absence of a control group, a larger sample size, and additional tests to rule out other causes of symptoms, such as milk protein intolerance.

10.1128/mbio.01051-22.2 TABLE S2 Summary of subjects’ self-reported symptoms during the HBT (a) and final week (b). Self-reported symptoms were quantified on a scale of 0 to 4, with 0 corresponding to no symptoms, 1 corresponding to very mild symptoms such as gassiness, 2 corresponding to mild to moderate symptoms such as brief cramping or other forms of abdominal pain, 3 corresponding to moderate symptoms such as cramping or other forms of abdominal pain for longer than half an hour, and 4 corresponding to severe symptoms such as diarrhea. Download Table S2, XLSX file, 0.01 MB. Copyright © 2022 Smith et al. 2022 Smith et al. https://creativecommons.org/licenses/by/4.0/ This content is distributed under the terms of the Creative Commons Attribution 4.0 International license.

10.1128/mbio.01051-22.5 FIG S3 Rating of self-reported symptoms by subjects (where 0 = no symptoms, and 4 = severe symptoms such as diarrhea) during each hydrogen breath test (HBT) versus the HBT area under the curve (AUC) of combined concentration for that HBT for each test for all subjects. No significant relationship was found overall (*r*[44] = 0.0915, *P* = 0.541). In addition, we did not observe a clear increase in symptom severity following the elimination phase (paired Wilcoxon signed rank test; *P* = 0.85) nor a statistically significant decrease in symptoms across subjects following the (re)introduction phase to baseline (paired Wilcoxon signed rank test; *P* = 0.42) as might have been expected from the increase in HBT AUC observed in Fig. 2. This may be attributable to the diverse range of possible symptoms and the challenge of quantifying them in a consistent and comparable manner across subjects, coupled with the relatively small number of subjects in the study. In addition, reports of symptom intensity can be subjective and can be influenced by subject perception, such as a placebo of expecting more symptoms the first HBT after not having had two cups of milk in a long time. In fact, the change in reported symptoms from the first to the second HBT was significant (paired Wilcoxon signed rank test; *P* = 0.036) and could reflect this placebo effect. Download FIG S3, TIF file, 1.5 MB. Copyright © 2022 Smith et al. 2022 Smith et al. https://creativecommons.org/licenses/by/4.0/ This content is distributed under the terms of the Creative Commons Attribution 4.0 International license.

### Subject identity determines microbiota structure over and above temporal factors, including dietary intervention.

**FIG 3:**
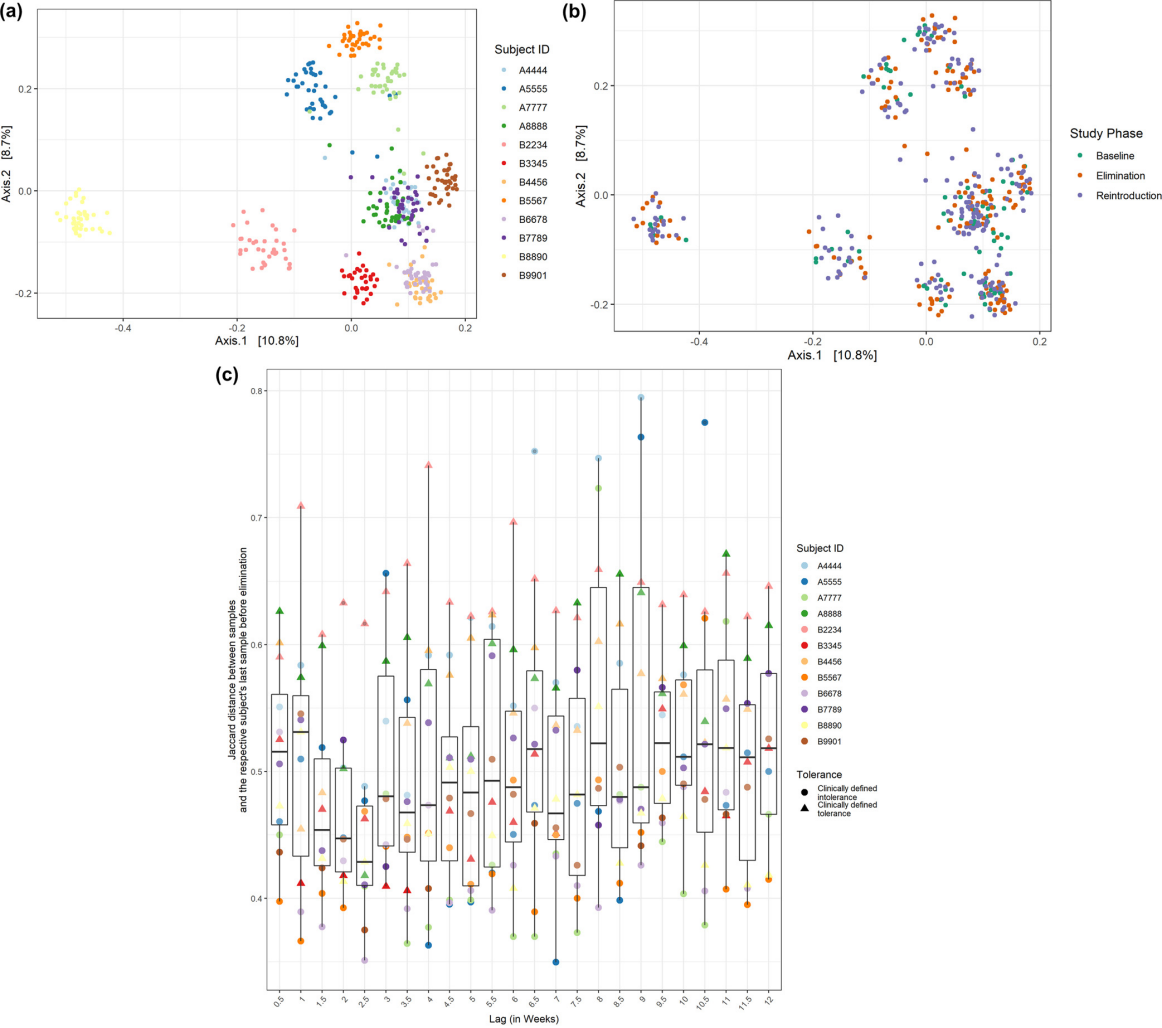
Individual overall microbiota structure was minimally perturbed by the dietary intervention. (a, b) Visualization of the first two principal coordinates from principal coordinate analysis (PCoA) across all samples for all subjects based on binary Jaccard, colored by subject (a) and by study phase (b). Samples throughout the study clustered predominately based on subject ID. (c) β-Diversity (binary Jaccard) between each subject’s last sample before dairy product elimination and that subject’s other time points. The half-week bins correspond to the number of days between when the samples in each sample pair were collected (lag), not the study day.

We next investigated changes in the gut microbiota structure of subjects throughout the study and in response to the dietary intervention using 16S rRNA gene sequencing. Fig. 3a and b display the first two coordinates of the principal coordinate analysis (PCoA) of all samples across time and all subjects, using binary Jaccard dissimilarity measures. Samples clustered predominantly by subject identity (Fig. 3a) over and above any clustering by study phase (Fig. 3b), indicating that individuality dominated any changes in microbiota structure over the course of the study (permutational multivariate analysis of variance [PERMANOVA] with 1,000 permutations on Jaccard dissimilarities; by subject identity: *R*^2^ = 0.758, *P* < 0.001; by study phase: *R*^2^ = 0.004, *P* = 0.707). Even when comparing the overall microbiota structure of an individual to themselves throughout the study, we did not identify any consistent shifts in microbial β-diversity or α-diversity in response to the elimination of dairy products or (re)introduction of whole cow milk (Fig. 3c). There was significant clustering of samples by study phase nested within each subject, but study phase explained only a small amount of variation in microbiota structure (PERMANOVA with 1,000 permutations on Jaccard dissimilarities by study phase nested within subject identity: *R*^2^ = 0.032, *P* < 0.001).

### Subtle microbiota changes in response to the dietary intervention are consistent across a few individuals.

The absence of a strong, consistent intervention-associated shift in overall subject microbiota structure did not rule out the possibility that the interventions had a more subtle effect, for example, on individual bacterial taxa. We next investigated whether there were specific taxa with a consistent shift in abundance across subjects. To identify such shifts between study phases, we performed linear discriminant analysis, a supervised method, using treeDA (29). This package performs discriminant analysis using a phylogenetic tree structure provided to the algorithm. The necessary inputs are the classes to be discriminated (i.e., elimination versus [re]introduction phase), a set of predictors (taxa abundances), and a phylogenetic tree describing the relationship between bacterial taxa. treeDA identified Bifidobacterium, Ruminococcus_2, and Agathobacter as key predictor genera that encompass many of the amplicon sequencing variants (ASVs) useful for distinguishing samples in the elimination phase from those in the (re)introduction phase. While the specific genera prioritized by treeDA were sensitive to the input parameters, this result suggested that there might be a consistent and interpretable microbiota structure shift across subjects (see Materials and Methods). The genus Bifidobacterium, for example, has previously been implicated in lactose tolerance, and many species within this genus have the ability to break down lactose (19, 28). In addition, previous studies have found that lactose consumption in non-lactase-persistent individuals is associated with increased abundance of Bifidobacterium in the intestinal microbiota (30, 31). However, increased Bifidobacterium abundance has also been implicated in lactose intolerance (32).

**FIG 4:**
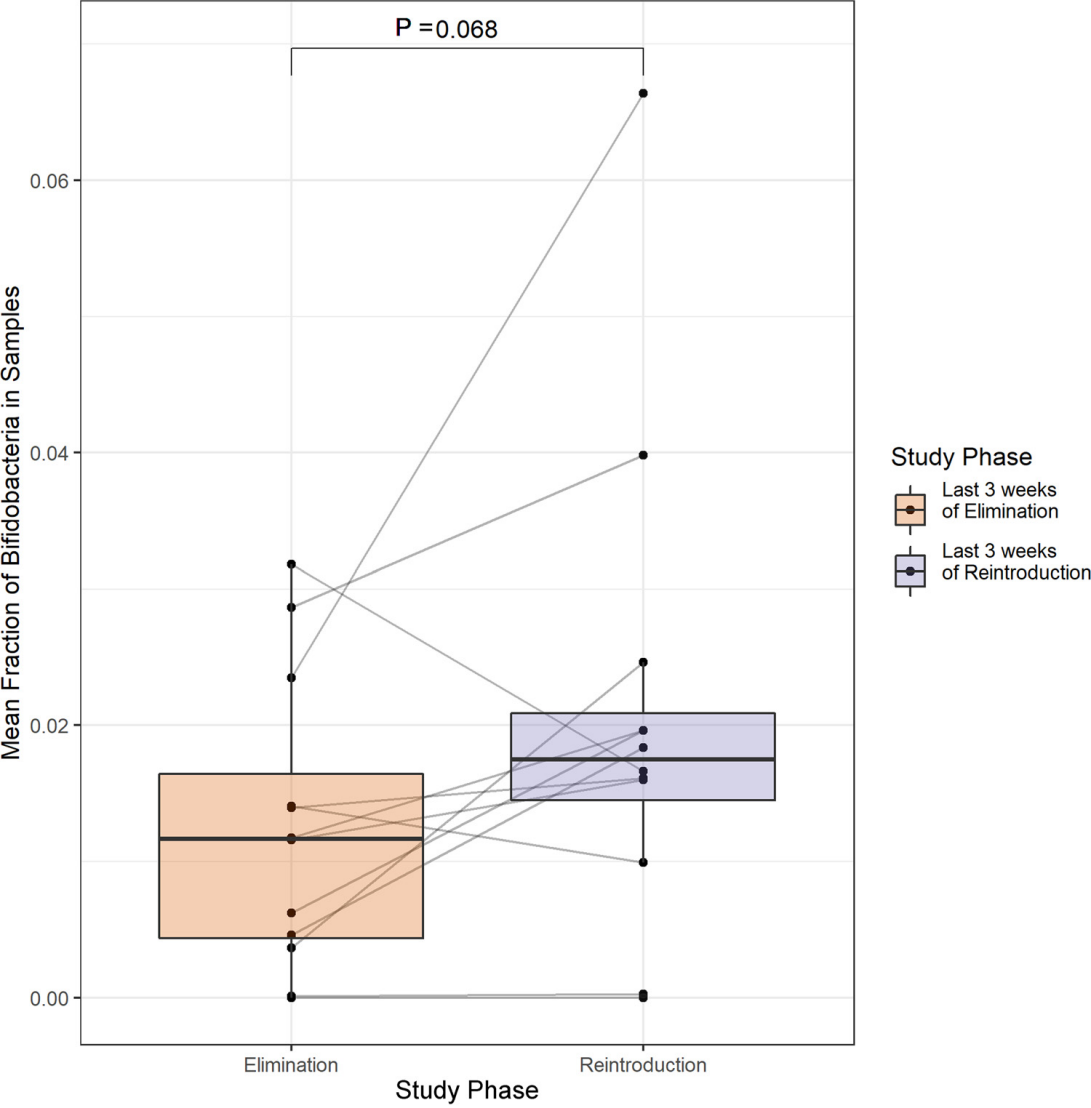
Positive but nonsignificant trend in relative Bifidobacterium abundance after whole cow milk (re)introduction. Mean fraction of Bifidobacterium in samples from the last 3 weeks of the elimination phase is compared to the mean fraction in samples from the last 3 weeks of the (re)introduction phase across all subjects.

Given this result, we investigated the change in abundance of ASVs assigned to the genus Bifidobacterium within subjects over the course of the entire study. Fig. S4 shows that there was no obvious decrease in the abundance of Bifidobacterium in most subjects during the elimination phase, nor an obvious increase during the (re)introduction phase as might have been expected (see Fig. S5 for results with other genera). There was a trend toward increased abundance of Bifidobacterium between the last 3 weeks of elimination and the last 3 weeks of (re)introduction in several subjects, but the increase in mean fraction of Bifidobacterium across all subjects from 0.0125 to 0.0206 was not statistically significant (paired Wilcoxon signed rank test; *P* = 0.068; Fig. 4). This result is consistent with prior studies that found a lack of consistent increase in Bifidobacterium abundance across participants in response to galactooligosaccharides, despite similar initial levels in responding and nonresponding individuals (33). Evaluation of the relationship between Bifidobacterium abundance and other lactose intolerance metrics can be found in Fig. S6 and S7; however, no significant correlation was found. For example, the correlation between the AUC of the HBT measurements and the abundance of Bifidobacterium was not significant (Spearman’s; *r* = −0.074, *P* = 0.625).

10.1128/mbio.01051-22.6 FIG S4 Abundance of Bifidobacterium in each sample (normalized based on the total bacterial abundance in that sample) for all subjects over the course of the study, colored by study phase. Bifidobacterium was one of the taxa prioritized by the supervised linear discriminant analysis with treeDA and was also one of our hypothesized candidate taxa. There was not an obvious decrease in the abundance of Bifidobacterium in most subjects during the elimination phase or an obvious increase during the (re)introduction phase as might be expected. Download FIG S4, TIF file, 2.3 MB. Copyright © 2022 Smith et al. 2022 Smith et al. https://creativecommons.org/licenses/by/4.0/ This content is distributed under the terms of the Creative Commons Attribution 4.0 International license.

10.1128/mbio.01051-22.7 FIG S5 Abundance of Ruminococcus_2 genus (a) and Agathobacter genus (b) in each sample (normalized based on the total bacterial abundance in that sample) for all subjects over the course of the study, colored by study phase. These genera, along with Bifidobacterium, encompass many of the amplicon sequencing variants prioritized by treeDA. There was not an obvious consistent shift in the abundance of Ruminococcus_2 *or* Agathobacter in most subjects during either the elimination or (re)introduction phase as might be expected. This may indicate that the prioritization from treeDA was a result of overfitting and not anything biological. Download FIG S5, TIF file, 1.8 MB. Copyright © 2022 Smith et al. 2022 Smith et al. https://creativecommons.org/licenses/by/4.0/ This content is distributed under the terms of the Creative Commons Attribution 4.0 International license.

10.1128/mbio.01051-22.8 FIG S6 The hydrogen breath test (HBT) area under the curve of combined concentration for all subjects together (a) and individually (b) versus the abundance of Bifidobacterium in the sample (normalized based on the total bacterial abundance in that sample) that was collected nearest in time to when the HBT was performed. No significant relationship was found (*r*[44] = −0.212, *P* = 0.152). Download FIG S6, TIF file, 1.4 MB. Copyright © 2022 Smith et al. 2022 Smith et al. https://creativecommons.org/licenses/by/4.0/ This content is distributed under the terms of the Creative Commons Attribution 4.0 International license.

10.1128/mbio.01051-22.9 FIG S7 (a) Clinical lactose tolerance status as defined by the hydrogen breath test (HBT) versus the abundance of Bifidobacterium in the sample (normalized based on the total bacterial abundance in that sample). (b) Rating of self-reported symptoms by subjects (where 0 = no symptoms, and 4 = severe symptoms such as diarrhea) during each HBT versus the fraction of Bifidobacterium in the sample nearest that HBT. The results did not show a significant relationship between abundance of Bifidobacterium (*t*[34] = −0.362, *P* = 0.719) and Clinical Tolerance or symptoms (*r*[44] = 0.035, *P* = 0.818). Download FIG S7, TIF file, 0.9 MB. Copyright © 2022 Smith et al. 2022 Smith et al. https://creativecommons.org/licenses/by/4.0/ This content is distributed under the terms of the Creative Commons Attribution 4.0 International license.

### Shifts in microbiota structure are tightly constrained and highly individualized even in the face of significant change in metabolic output.

Fig. S8 shows a weak correlation between the first principal coordinate from the PCoA displayed in Fig. 3a and time. We wondered whether individuals with greater gut microbiota variability over time had greater HBT variability among tests. To evaluate this, we computed the correlation between microbiota dispersion, as measured by average Bray-Curtis distance to the median, and the variance of HBT AUCs; the correlation was not significant (Spearman’s; *r* = 0.343, *P* = 0.275).

10.1128/mbio.01051-22.10 FIG S8 Visualization of the first principal coordinates from principal coordinate analysis (PCoA) across all samples for each subject individually using Bray-Curtis (a, c, e) and binary Jaccard (b, d, f) versus the day in the study for that subject that the sample was collected, colored by study phase. These results suggest that there is little correlation between the first principal coordinates from the PCoAs in Fig. 3 and the time course of the study. It is surprising that even for nonweighted distance measures such as binary Jaccard, there seem to be limited overall microbiota changes, which means that few low-abundance species, such as the candidate taxa expected to respond to the intervention, dropped in or out of presence in the microbiota. A caveat to this, however, is that the interventions might not have been dramatic or long enough and that if they were, a shift would have been found. Download FIG S8, TIF file, 2.8 MB. Copyright © 2022 Smith et al. 2022 Smith et al. https://creativecommons.org/licenses/by/4.0/ This content is distributed under the terms of the Creative Commons Attribution 4.0 International license.

We did not find evidence that temporal dynamics or dietary variability were major determinants of variation in microbiota structure; on the contrary, there were surprising levels of resistance to perturbation in microbiota structure. An individual’s microbiota structure can be viewed as located in a multidimensional space that describes the possible variation in microbiota structures. A conservative proxy for the range of structures within this space that are feasible among healthy individuals is the range spanned by the samples of all subjects in the study. Fig. 3 suggests that each individual’s microbiota is individualized throughout the study and tightly constrained to a small subsection within this range. The individualized nature of the microbiota recapitulates findings of many previous studies (34–42). The resilience of human microbiotas to perturbations has also been reported extensively (35, 43, 44) but contrasts with some studies of diet shift that triggered significant microbiota alterations (45–48). To probe this discrepancy, we quantified the dynamics of change over time in each individual’s microbiota.

Specifically, we investigated microbiota *autocorrelation time*, i.e., the time interval between two samples from a subject across which the difference in microbiota structure approaches the difference between randomly chosen pairs of samples from that individual. We defined autocorrelation time as the typical number of days that it takes until two consecutive samples from the same subject have the same distance between them as equal to 90% of the median distance between pairs of randomly chosen samples from that subject.

**FIG 5:**
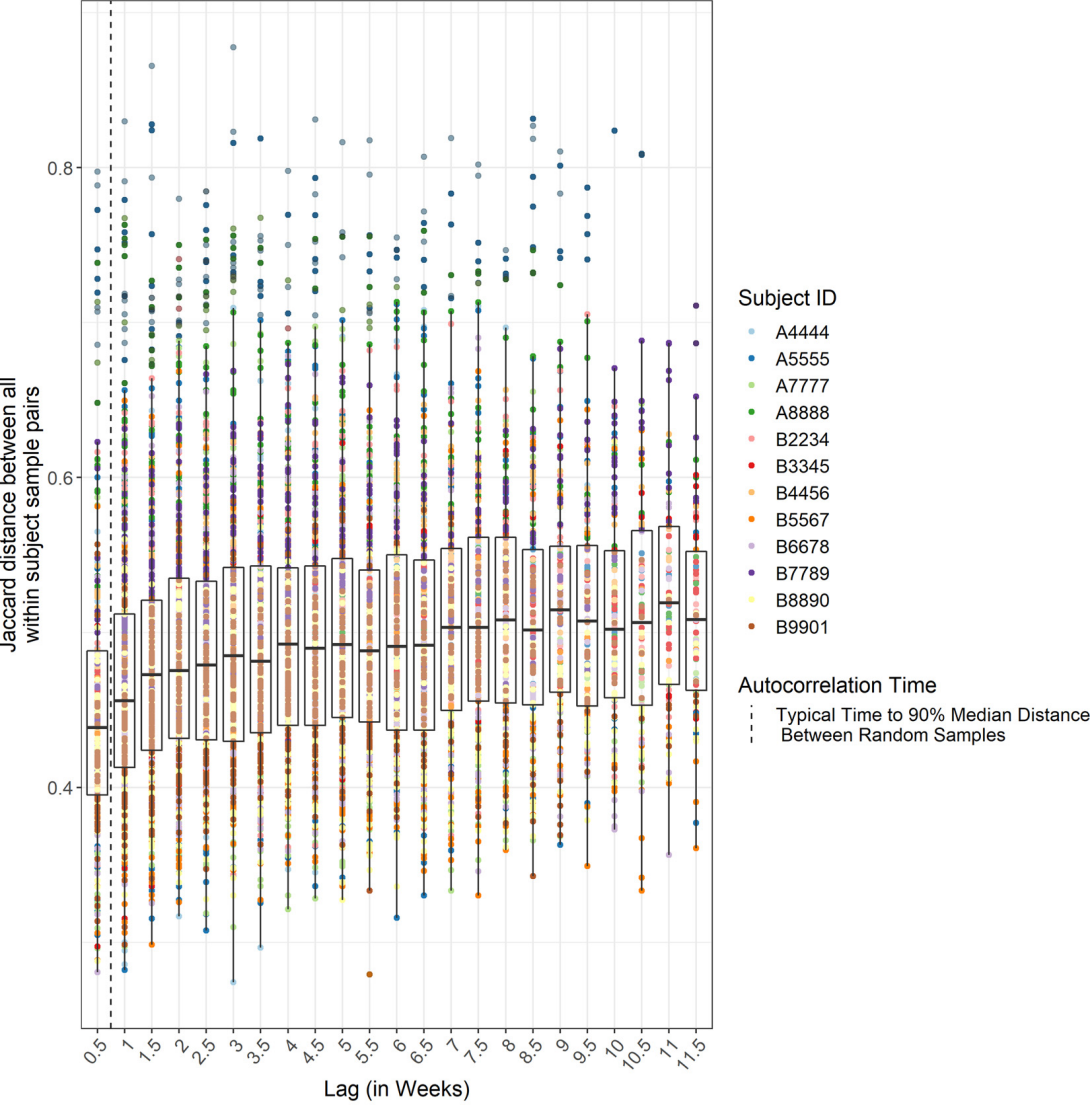
β-Diversity of microbiota shows rapid change over time. Binary Jaccard between every sample pair from the same subject throughout the study is plotted against time interval in weeks.

We found an autocorrelation time of 5.25 days (SD ± 2.72 days) using binary Jaccard as the distance metric. Thus, the typical change in microbiota structure during a week approaches the median change between any two time points during the course of the study (see Materials and Methods for comparison with same-day control swabs). This rapid change in microbiota structure *within* each individual contrasts sharply with the restricted and stable nature of individualized variation. Importantly, these findings applied both within and across study phases, highlighting the resistance to perturbation of each individual’s microbiota structure (Fig. 5). Pairs of samples collected over longer periods of time than the autocorrelation time showed little to no correlation between the binary Jaccard dissimilarity and the number of days that separated them.

## DISCUSSION

We have investigated how self-reported lactose intolerance symptoms, lactose intolerance clinical diagnostic test results, and gut microbiota structure vary in response to complete lactose-containing dairy product elimination and subsequent milk (re)introduction. We found that 8 of 12 subjects were clinically lactose intolerant at baseline and remained that way throughout the study. Of note, 10 of 12 subjects had only mild or no symptoms while drinking two cups of milk per day by the end of the milk (re)introduction period. This is consistent with prior literature that reported examples of subjects tolerating two cups of milk with only mild symptoms despite being defined as clinically lactose intolerant by the HBT (49). The discrepancy between the HBT results and the self-reported symptoms suggests that the clinical test used to diagnose lactose intolerance might tell only part of the story. However, a weakness of our study was that self-reported symptoms were not evaluated in a framework involving a control group, a larger number of subjects, and tests to rule out other causes of symptoms. Therefore, in the future, it could be useful to investigate the comparative efficacy of different dietary interventions for patients experiencing symptoms of lactose intolerance after dairy product consumption.

In addition, the increase in HBT AUC suggests that 4 weeks of complete dairy product elimination might increase intolerance. The HBT AUC offers an interesting alternative for the classification of lactose intolerance, because it offers more nuanced information than the binary results of the current clinical assessment. Although we report a potentially meaningful trend in the results, further research will be needed to understand better the utility of this metric and the nature of its relationship to symptoms.

The gut microbiota is often described as constantly changing, on a broad range of time scales, both in response to a plethora of external factors and as a result of random species’ drift (46, 50). Previous studies have indicated that dietary interventions may cause dramatic shifts in microbiota structure (46, 51–54). Our results stand in contrast to these previous observations: in our study, a relatively dramatic dietary change—(re)introduction of lactose-containing dairy products, even as much as two glasses of whole cow milk per day, after complete dairy product elimination—caused surprisingly limited change in microbiota structure. In particular, this change did not draw the subjects’ microbiota structures beyond the regions that they occupied in the space of possible microbiota structures prior to the perturbation, and it did not even limit the compositions of microbiota structures to subsections within these regions. Each subject’s fecal microbiota remained highly individualized, and pairs of samples from the same individual collected months apart were as similar as pairs of samples collected less than a week apart.

Determining whether and how gut microbiotas from different regions of the intestinal tract resist (or respond to) different types of dietary manipulations will be key to understanding the dynamics of the gut ecosystem and to designing personalized microbiota-directed interventions. One possible reason for contrasting results might be related to the nature of the dietary change, possibly including both the subtlety of the dietary intervention and the nutrient categories affected (53). For example, manipulation of fiber intake, a major influence on the nutrient profile that reaches the distal gut, might have a greater impact on fecal microbiota structure than dietary changes that focus on other components, such as lactose-containing dairy products (55, 56). Some dietary interventions may indirectly affect nutrient categories other than that being directly manipulated, such as an increase in fiber resulting from elimination of meat products and a subsequent increase of plant products in their place (57). The discrepancies in the extent and nature of microbiota changes caused by dietary interventions found among different studies, even dietary interventions that we perceive as similarly impactful, highlight the need for a mechanistic understanding of the drivers of change in these complex systems and the necessity for a better understanding of the ecological principles that underlie the structure of the microbiota.

Our study was limited by the insensitivity of fecal samples to microbial community responses confined to the lower small intestines and proximal colon, which may be undetected or obscured by the lack of change in other regions of the colon, and by the inability of 16S rRNA-based analyses to detect changes in microbial metabolic activity (46) and protein expression. Future studies of the effects of dairy products on the gut microbiota would benefit from larger cohorts, longer elimination and reintroduction phases, specimens from the small intestines and proximal colon, and other types of microbiota and host-based measurements (54) (see the supplemental materials for additional lessons and future directions).

The difference between the effects of our dietary intervention and those in other studies also hints at the possibility that there may have been some publication bias with respect to reports about drivers of change in microbiota structure: interventions resulting in little change in microbiota structure may be less likely to be published. Our findings emphasize that publication of such results, often viewed as “negative results,” is crucial to making progress in the understanding of microbiota dynamics and its response to external factors.

To the best of our knowledge, this is the first study of human microbiota survey data alongside a noninvasive quantitative biochemical assay to assess the effect of a deliberate dietary intervention on the distal gut microbiota structure and host phenotype. Despite the surprising level of resistance to perturbation in response to this intervention in terms of clinically defined lactose tolerance status and of fecal microbiota structure, future studies will benefit from the coupling of such complementary approaches to probe how interventions designed to manipulate the microbiota may influence phenotypic traits of the host, especially those relevant to human health.

## MATERIALS AND METHODS

### Ethics statement.

The research was approved by an Administrative Panel for the Protection of Human Subjects (Institutional Review Board) of Stanford University (protocol 42241). All subjects were properly informed of the risks and benefits of this study and then signed an approved, written consent form.

### Experimental design.

The response of the human gut microbiota to lactose-containing dairy product elimination and (re)introduction was evaluated by collecting swabs of fecal samples three times per week from 12 healthy subjects for 12 weeks. The 12 weeks were divided into three dietary phases: 2 weeks of normal diet, which is referred to as “baseline”; 4 weeks of complete dairy product elimination, referred to as “elimination”; and 6 weeks of controlled gradual (re)introduction of whole cow milk, referred to as “(re)introduction.” Because some subjects ingested no lactose-containing products at baseline, this third phase could be viewed as an “introduction” to these products. Details of the (re)introduction protocol can be found in Fig. 1. All subjects used the same type and brand of whole cow milk throughout the study.

Clinical tolerance of lactose was evaluated with a HBT, which is the clinical standard for the evaluation of lactose intolerance, before and after each diet phase (Fig. 1). Twenty-four hours before each HBT, the subjects followed a strict diet and fasting protocol as commonly used in clinical practice in preparation for the HBT to limit foods that may linger and produce delayed gas. On the morning of each HBT, subjects collected a single breath sample using an at-home collection kit from QuinTron Instrument Company (West Milwaukee, WI). They then drank two cups of whole cow milk. At every half hour over the next 6 h, they collected an additional breath sample and recorded the severity and type of symptoms they experienced, when applicable. Throughout the study, subjects recorded all dairy product consumption, major deviations in lifestyle, lactose intolerance symptoms, and any compliance issues. Two aspects of this HBT protocol differed from the standard procedure in the clinic. First, the duration of the sample collection period was extended to 6 h from the standard of up to 3 h, motivated by our interest in quantifying lactose intolerance beyond a binary classification of intolerant or not. Second, two cups of whole cow milk were used in place of the standard procedure of adding 25 g of lactose powder (approximately equivalent to the amount of lactose in 2 cups of milk) to water to evaluate lactose intolerance in the context of dairy products.

DNA was extracted from the stool samples, PCR amplified, and used for amplicon sequencing of the V4 to V5 region of the 16S rRNA gene. The data were analyzed to reveal community structure, in an effort to characterize one aspect of gut microbiota response to dairy product elimination and (re)introduction, and to assess the correlation of microbiota structure with changes in lactose intolerance as reflected by the HBT and reported symptoms. The HBT and reported symptoms were also analyzed to assess the response to this dietary intervention. Self-reported symptoms were quantified on a scale of 0 to 4, with 0 corresponding to no symptoms, 1 corresponding to very mild symptoms such as gassiness, 2 corresponding to mild to moderate symptoms such as brief cramping or other forms of abdominal pain, 3 corresponding to moderate symptoms such as cramping or other forms of abdominal pain for longer than half an hour, and 4 corresponding to severe symptoms such as diarrhea.

### Subjects and sampling protocol.

Healthy nonpregnant adults with self-reported mild lactose intolerance were recruited from the Stanford community and nearby area, excluding individuals with chronic disease, hospitalization or antibiotic use in the previous 6 months, immunizations, or international travel in the previous 4 weeks, or routine use of any prescription medication except birth control or hormone replacement therapy. Characteristics of the 12 subjects who completed the sampling protocol are summarized in Table S1. Subjects collected two swabs of each stool sample at home, which were frozen immediately in *RNAlater* (Sigma-Aldrich, St. Louis, MO) in home freezers. The samples were transferred without thawing to −80°C storage in the laboratory approximately within a week of when subjects completed the study.

A total of 1,008 stool swab samples were collected; the timing of samples throughout the study for each subject is shown in Fig. 1. Some intended daily samples were not collected because subjects did not produce stool that day, in which case samples were collected at the next stool sample opportunity. Sixteen subjects enrolled in and began the study, but four subjects did not complete the full dietary interventions due to reasons unrelated to the study and were thus excluded from analysis.

### Sample processing and DNA extraction.

All chemicals, solvents, and reagents were purchased from Sigma-Aldrich unless otherwise noted. Half of the collected swab samples, one of the two swab samples collected each collection day, were processed for DNA sequencing, and the other half remained at −80°C and were used as backup in case of contamination during the sample preparation process. Swab samples were thawed to room temperature during a 10-min centrifugation at 6,000 × *g* and then transferred to bead tubes from the MP Biomedicals (Irvine, CA) lysing matrix E kit. Extraction was then performed with the DNA/RNA 96 kit (Qiagen AllPrep; Germantown, MD) following the manufacturer’s protocol after homogenization for 1 min at speed 6.5 using MP Biomedicals FastPrep-24 5G Instrument followed by incubation for 5 min at 4°C and 10 min of centrifugation at 15,000 rpm. Five extraction control blanks were included per extraction plate.

### 16S rRNA gene sequencing.

Primers were purchased from Integrated DNA Technologies (Coralville, IA). The V4 to V5 region of the 16S rRNA gene was amplified for sequencing using 515F and barcoded 926R primers (515F forward primer: GTGYCAGCMGCCGCGGTAA; 926R reverse primer: CCGYCAATTYMTTTRAGTTT) (58). Triplicate 25-μL PCRs using Hot MasterMix (5 Prime) with 2 μL extracted DNA as the template and 10 μg/μL bovine serum albumin (BSA) were cycled as follows: denaturation at 94°C for 3 min, 25 cycles of 94°C for 45 s, 52°C for 60 s, 72°C for 120 s, and final extension at 72°C for 10 min. PCR amplicon libraries were purified using the UltraClean-htp 96-well PCR cleanup kit (Qiagen). Amplicon libraries were quantified by fluorometry (Quant-iT dsDNA high-sensitivity kit; Invitrogen, Waltham, MA) on a SynergyHT plate reader (BioTek) and combined in equimolar ratios into one pool. The pooled library was concentrated by ethanol precipitation and gel purified (QIAquick gel extraction kit; Qiagen). Each pool of V4 to V5 16S rRNA amplicons was sequenced (2 × 300-nt paired end) on one lane of a MiSeq V2 sequencer (Illumina, San Diego, CA) at the Carver Biotechnology Center of the University of Illinois, producing an average of 42,673 reads/sample, with a total of 24,579,392 reads produced for this study. Raw reads were demultiplexed using QIIME 1 (version 1.9.1) (59), trimmed of nonbiological sequence using cutadapt (version 1.14) (60), and resolved into ASVs using DADA2 (version 1.1) (61). Taxonomy was assigned using a SILVA reference database (version 132) (62) and DADA2 implementation of the RDP naive Bayesian classifier (63). A phylogeny was built using a SILVA backbone tree (version 132) (62) and the QIIME 2 fragment insertion plugin (64), which runs SEPP (65).

### HBT processing.

At-home HBTs were conducted before and after each of the three study phases for four tests in total for each subject (Fig. 1). Thirteen breath samples were collected for each HBT using the at-home QuinTron EasySampler breath collection kit. Breath samples were processed within 14 days of sample collection using the QuinTron BreathTracker analyzer and AlveoVac extraction system at the Stanford Digestive Health Center in Redwood City, California, to measure the concentrations of hydrogen, methane, and carbon dioxide in each sample. Each test was first analyzed using the clinical definition of lactose intolerance, which is an increase greater than 20 ppm combined concentration of hydrogen and methane gas in any breath sample above the concentration in the baseline sample. The AUC for each test was calculated by first subtracting the combined concentration of hydrogen and methane gas in each sample from the combined concentration of hydrogen and methane gas in the baseline sample, then plotting this adjusted concentration for each of the 13 breath samples for each test versus the minutes elapsed from the first sample, and taking the integral of the line connecting the data points. For some tests, the baseline sample had a concentration above that of breath samples collected later in the study, producing negative adjusted values. A negative concentration is not biologically interpretable, so for tests that had a later breath sample less than the baseline sample concentration, we set all concentrations that would have been negative to 0. The raw AUC results, including the negative values, are shown in Fig. S2. Two subjects during the first baseline HBT completed the full HBT procedure but did not properly collect breath samples, such that the concentrations of hydrogen and methane in the breath sample were not able to be determined. Thus, no HBT data for these two tests were included in any plots or analyses.

### Principal coordinate analysis.

Throughout our analysis, both binary Jaccard and Bray-Curtis dissimilarity measures were used. Whereas Bray-Curtis considers both structure and abundance, binary Jaccard only takes into account the presence or absence of a given taxon. This allowed us to detect changes in the abundance of taxa overall, as well as the appearance or disappearance of taxa, particularly low-frequency species. Both measures demonstrated similar results, so only plots using binary Jaccard were included in the main text, and the corresponding Bray-Curtis plots were included in the supplemental figures. Fig. 3C shows the β-diversity (binary Jaccard) between each subject’s last sample before dairy product elimination and that subject’s other time points. The samples were assigned to half-week bins with a maximum of one sample from each subject included in each bin. If more than one sample for a given subject were collected within a given half-week bin, one sample was randomly selected as the one to be included. Not every bin had a sample from every subject due to occasional incidental variations in sampling frequency by some subjects.

### Linear discriminant analysis.

We performed a supervised linear discriminant analysis using treeDA (29). This package performs sparse discriminant analysis using a phylogenetic tree structure provided to the algorithm. Incorporating the phylogenetic tree structure into the sparse discriminant analysis expands the feature space to include higher-order taxonomic units as predictors alongside the specific ASVs. For example, it is possible that at the ASV level, a signal cannot be detected, due to high strain-level variation between participants, but a consistent pattern can be seen across participants when considering a higher taxonomic level such as with the family Bifidobacteriaceae. The necessary inputs are the classes to be discriminated (our elimination versus [re]introduction phase samples), a set of predictors (the taxa abundances identified in our data), and a tree describing the relationship between the predictors (the phylogenetic tree of the microbial taxa). We compared samples from the last 3 weeks of the elimination phase to those in the last 3 weeks of the (re)introduction phase, so that the intervention had time to take effect, especially during the (re)introduction, which was a more gradual intervention. This resulted in a confusion matrix that correctly labeled 91 of the 105 elimination samples as elimination, and 85 of the 106 (re)introduction samples as (re)introduction. We also compared samples from the entire elimination phase versus those from the entire (re)introduction phase, as well as those from just the last 2 weeks of the elimination and (re)introduction phases. Of note, the resulting top taxa prioritized by this analysis were highly sensitive to parameter input, such as transformation and filtering of sample counts, and were not all supported by some of the further investigations.

We evaluated the performance of treeDA on a negative control, consisting of a test discriminating among samples across the entire study based on whether a sample was processed on an even or odd plate, which should have no correlation with microbiota structure. This control test resulted in a confusion matrix that incorrectly classified the plate as even for 150 of the 172 samples on an odd-numbered plate and classified the plate as odd for 210 of 232 samples on even-numbered plates, indicating that the model was, as expected, unable to discriminate between these arbitrary classifications relative to microbiota structure and was instead simply classifying almost all samples as even. We also repeated the test to discriminate between samples in the last 3 weeks of the elimination phase versus the last 3 weeks of (re)introduction phase on each subject individually to see—if we intentionally overpowered the test—whether it could find any signal, but this did not result in any interpretable consistent patterns.

### Autocorrelation time.

We defined autocorrelation time as the typical number of days that it takes until two consecutive samples from the same subject have at least the same distance between them as 90% of the median distance between any two randomly chosen samples from the same subject. To calculate this, we first determined the median distance across all sample pairs from the same subject regardless of the days separating the two, giving the median distance between any two randomly picked samples for a given subject. Then, we binned each sample pair from the same subject into half-week bins (based on the days separating collection of the samples) and calculated the median distance for each half-week bin for each subject. We then found, for each subject, the minimum half-week bin whose median distance between samples reached at least 90% of the distance between the median distance between any two randomly picked samples for that subject. We took the median of this number across subjects giving us an autocorrelation time of 5.25 days using the binary Jaccard measure. In addition, we calculated the autocorrelation time using Bray-Curtis, which resulted in an autocorrelation time of 7.00 days.

The choice of defining autocorrelation as a fraction of the median distance between any two randomly chosen samples was to account for the expected asymptotic nature of the distance between microbiota samples over time. Although we reported the autocorrelation time defined using a fraction of 90%, this fraction choice was arbitrary, so we also calculated the autocorrelation time based on definitions using 80 and 95%. The former gave an autocorrelation time of 3.50 days, and the latter gave an autocorrelation time of 10.5 days.

The median value across subjects of 90% of the median distance between any two randomly chosen samples (for each subject) was 0.394 for binary Jaccard and 0.310 for Bray-Curtis. This was higher than the average median distance between our technical controls (five pairs of two swabs from the same stool) of 0.267 for binary Jaccard and 0.152 for Bray-Curtis.

### Data availability.

Reads were deposited in SRA under BioProject PRJNA823665. The analysis code is available at https://github.com/courtrun/LactoseIntoleranceGutMicrobiota/tree/main.
