# Supplementary figures and images for: Short-Term Dairy Product Elimination and Reintroduction Minimally Perturbs the Gut Microbiota in Self-Reported Lactose-Intolerant Adults
Source: mBio. 2022 Jun 13;13(3):e01051-22. doi: 10.1128/mbio.01051-22 (PMC9239098; doi:10.1128/mbio.01051-22)

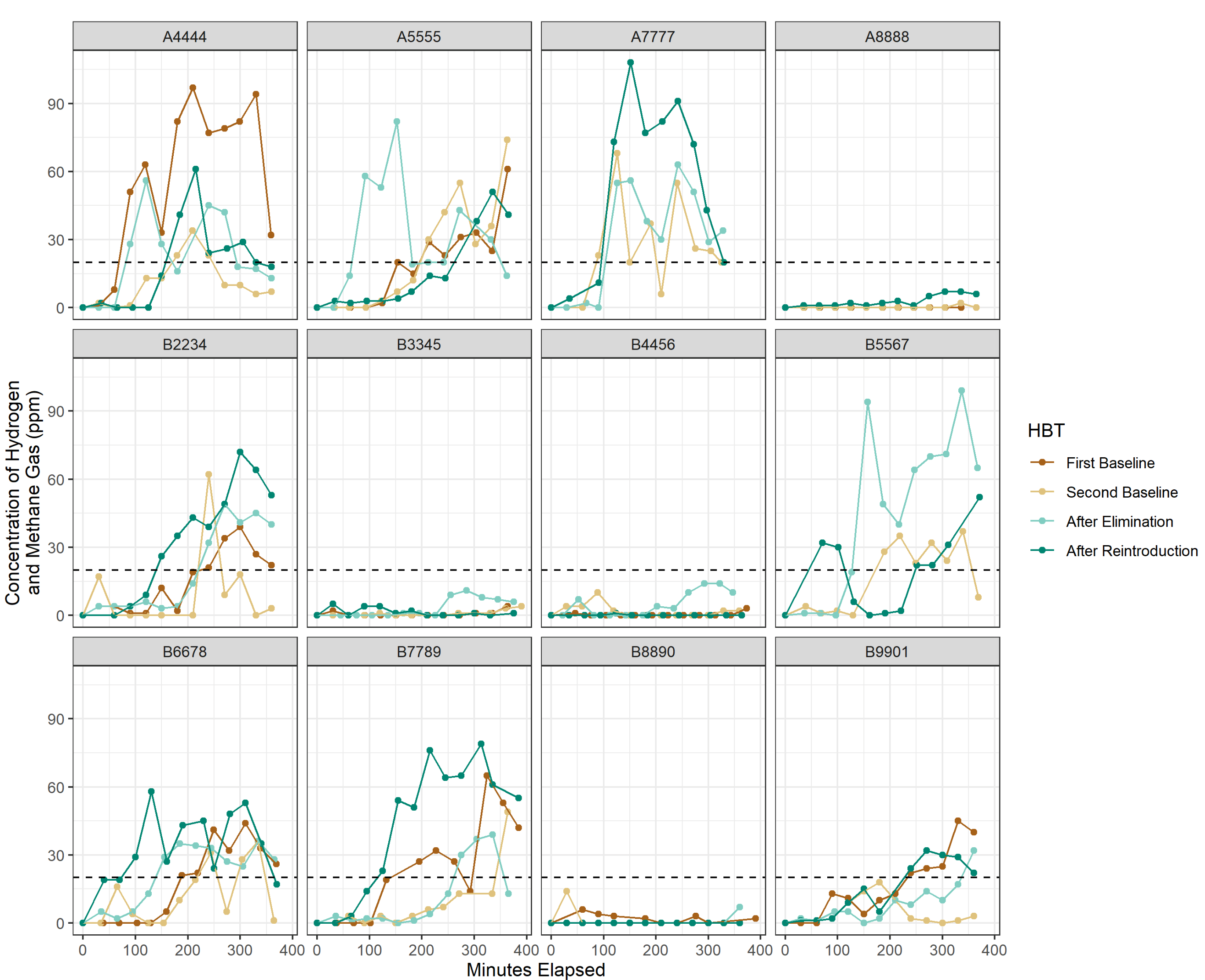

Supplement: FIG S1 [file mbio.01051-22-s0003.tif]

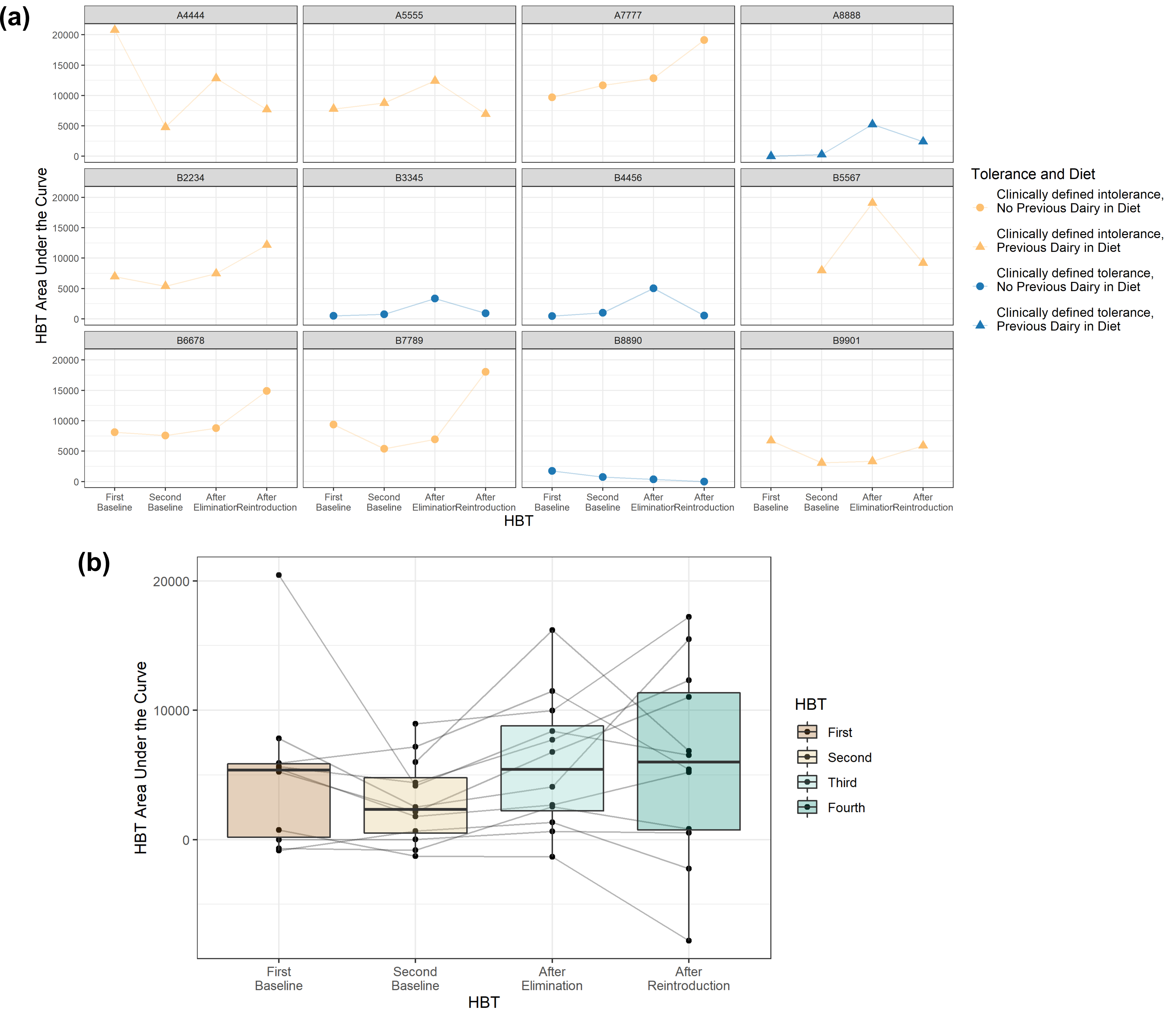

Supplement: FIG S2 [file mbio.01051-22-s0004.tif]

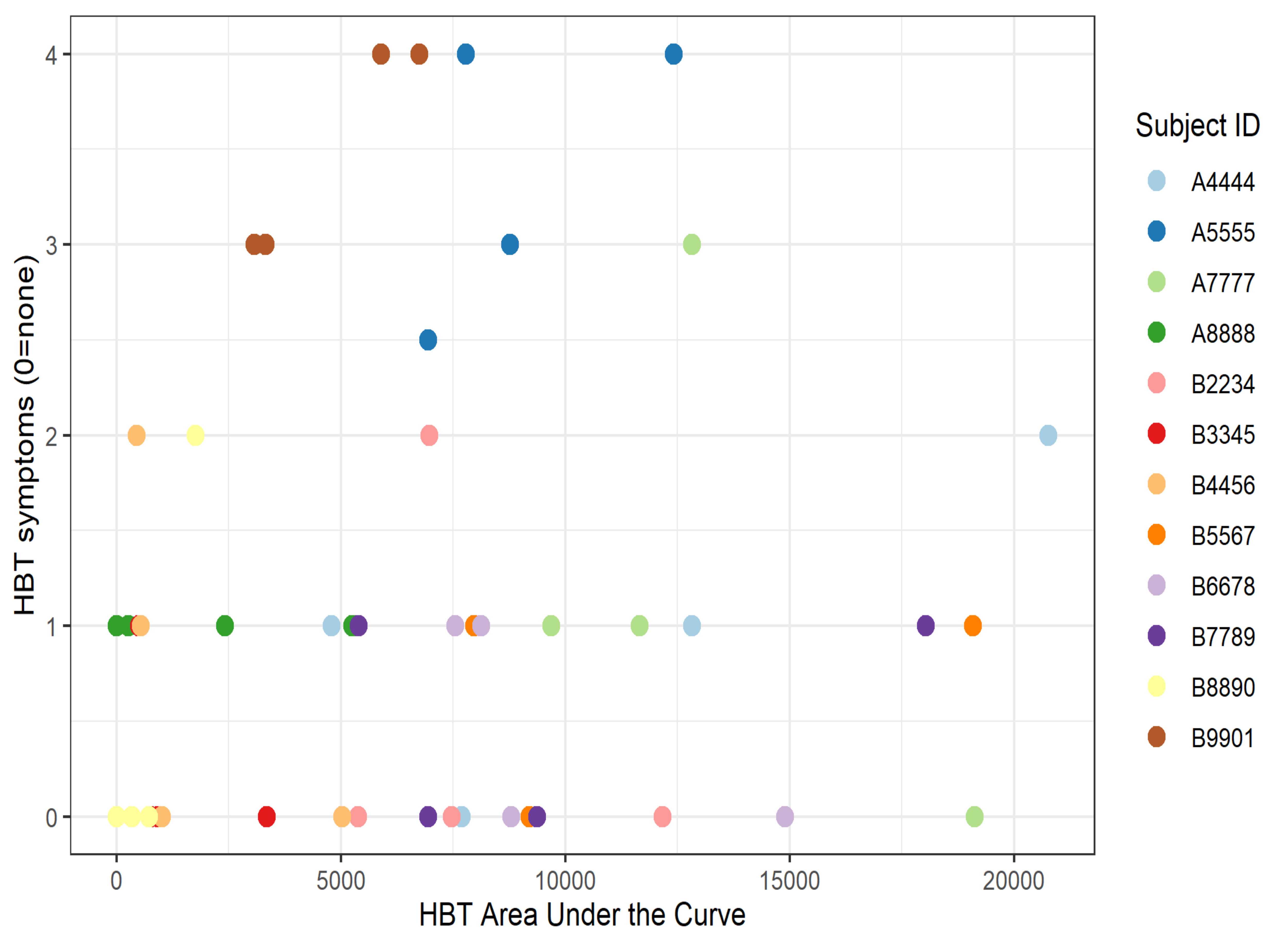

Supplement: FIG S3 [file mbio.01051-22-s0005.tif]

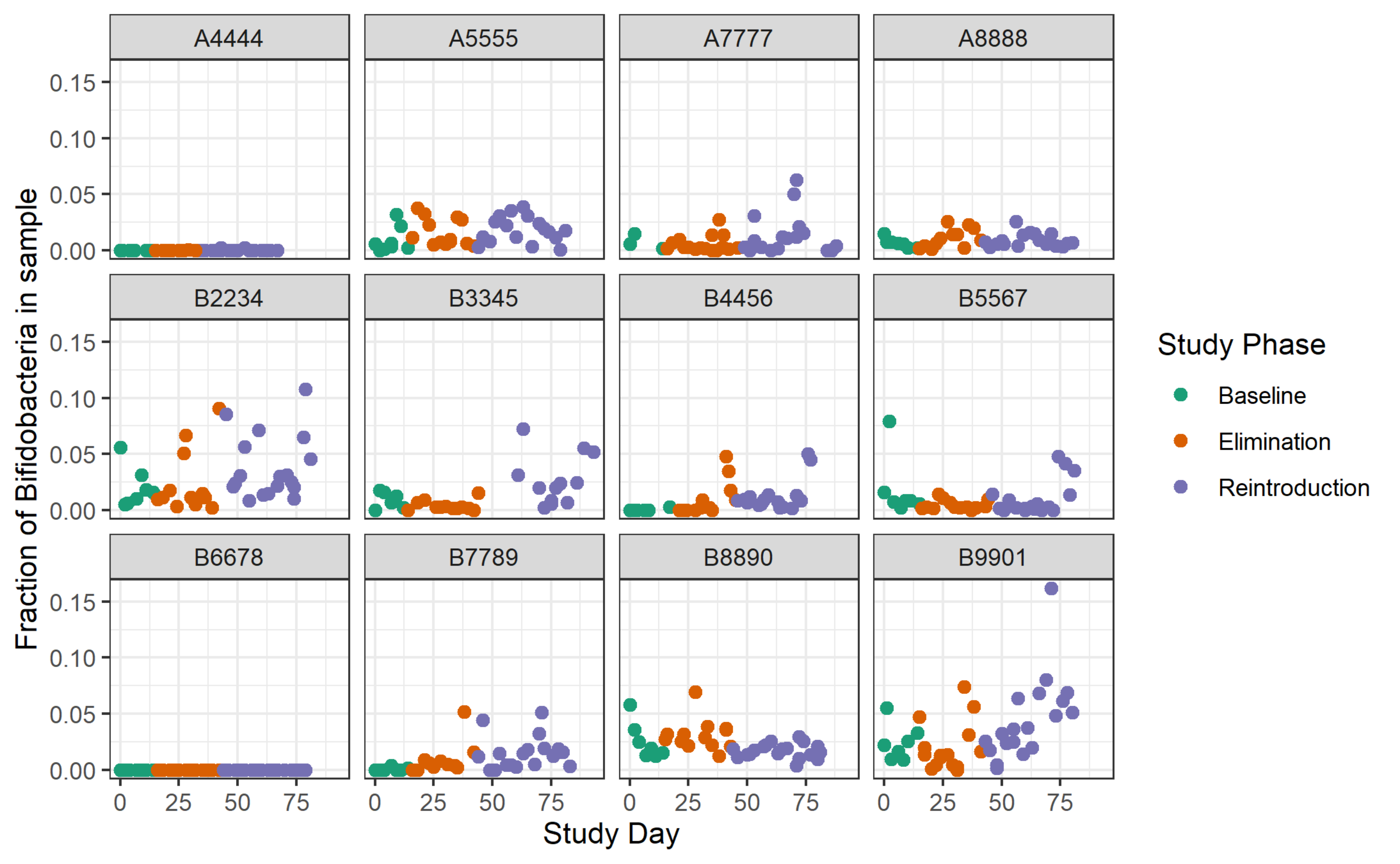

Supplement: FIG S4 [file mbio.01051-22-s0006.tif]

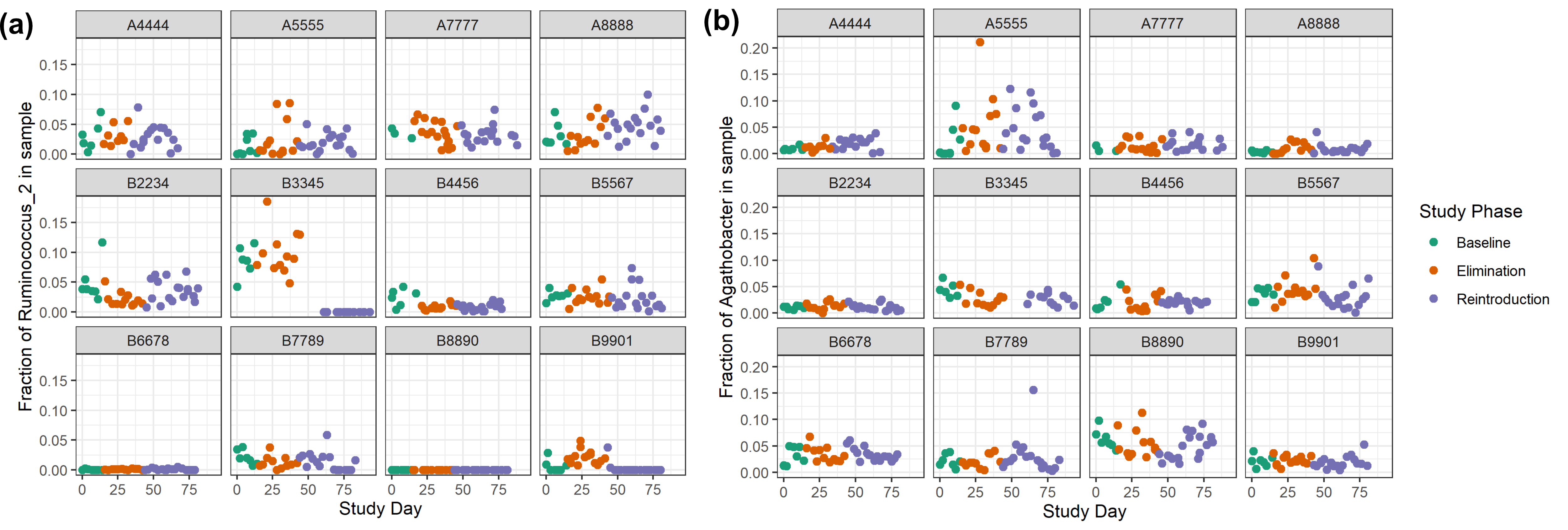

Supplement: FIG S5 [file mbio.01051-22-s0007.tif]

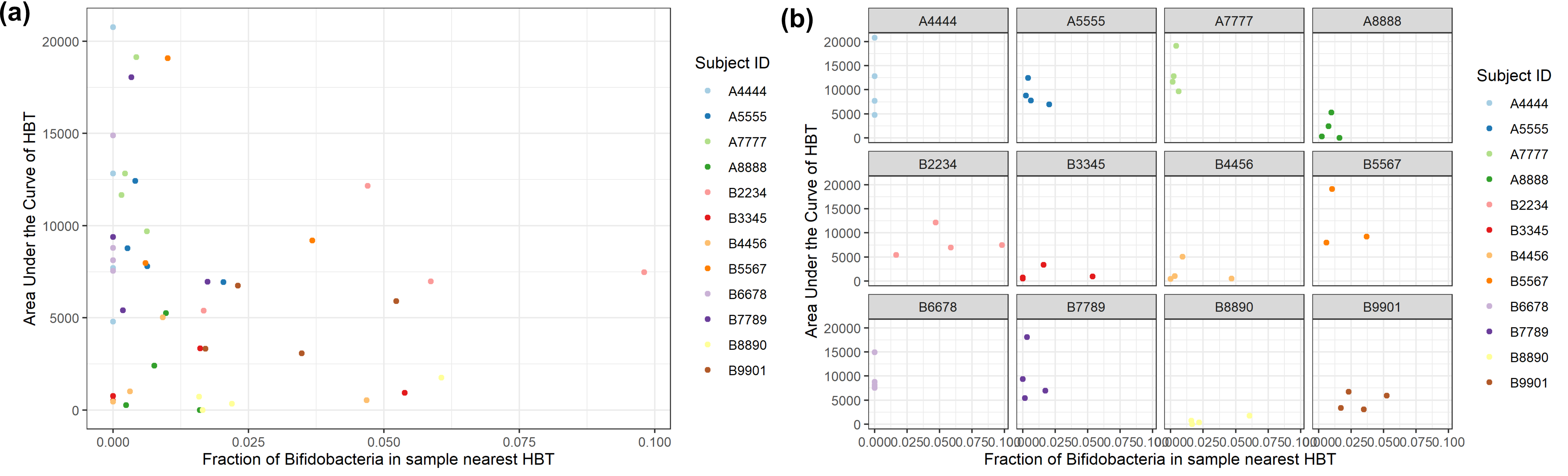

Supplement: FIG S6 [file mbio.01051-22-s0008.tif]

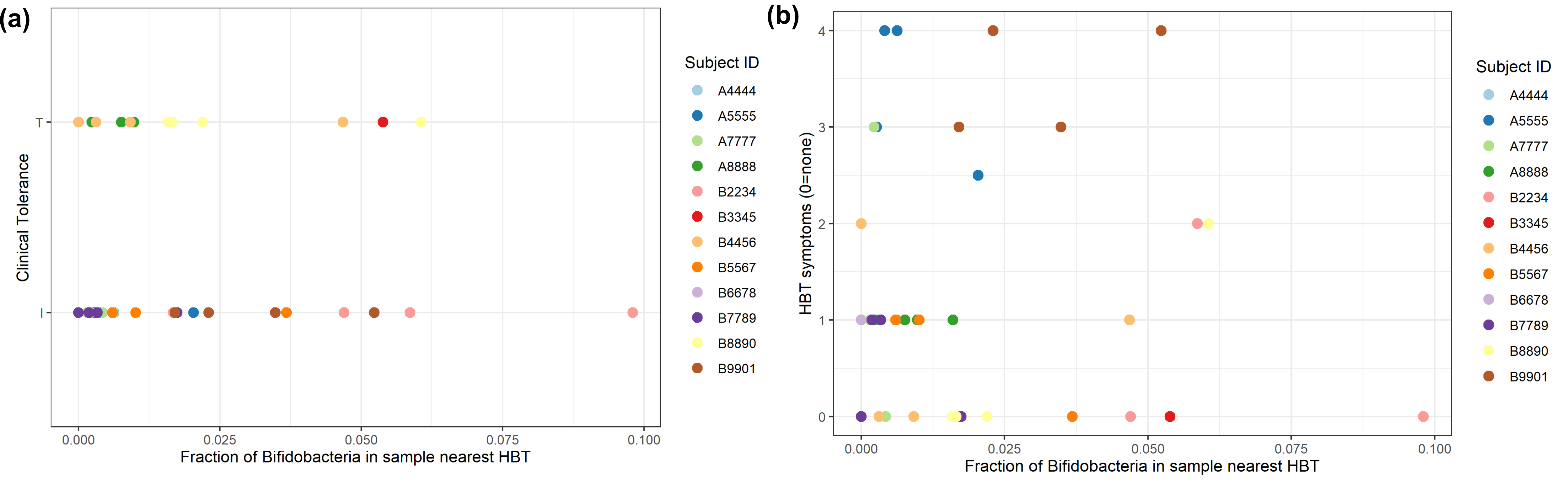

Supplement: FIG S7 [file mbio.01051-22-s0009.tif]

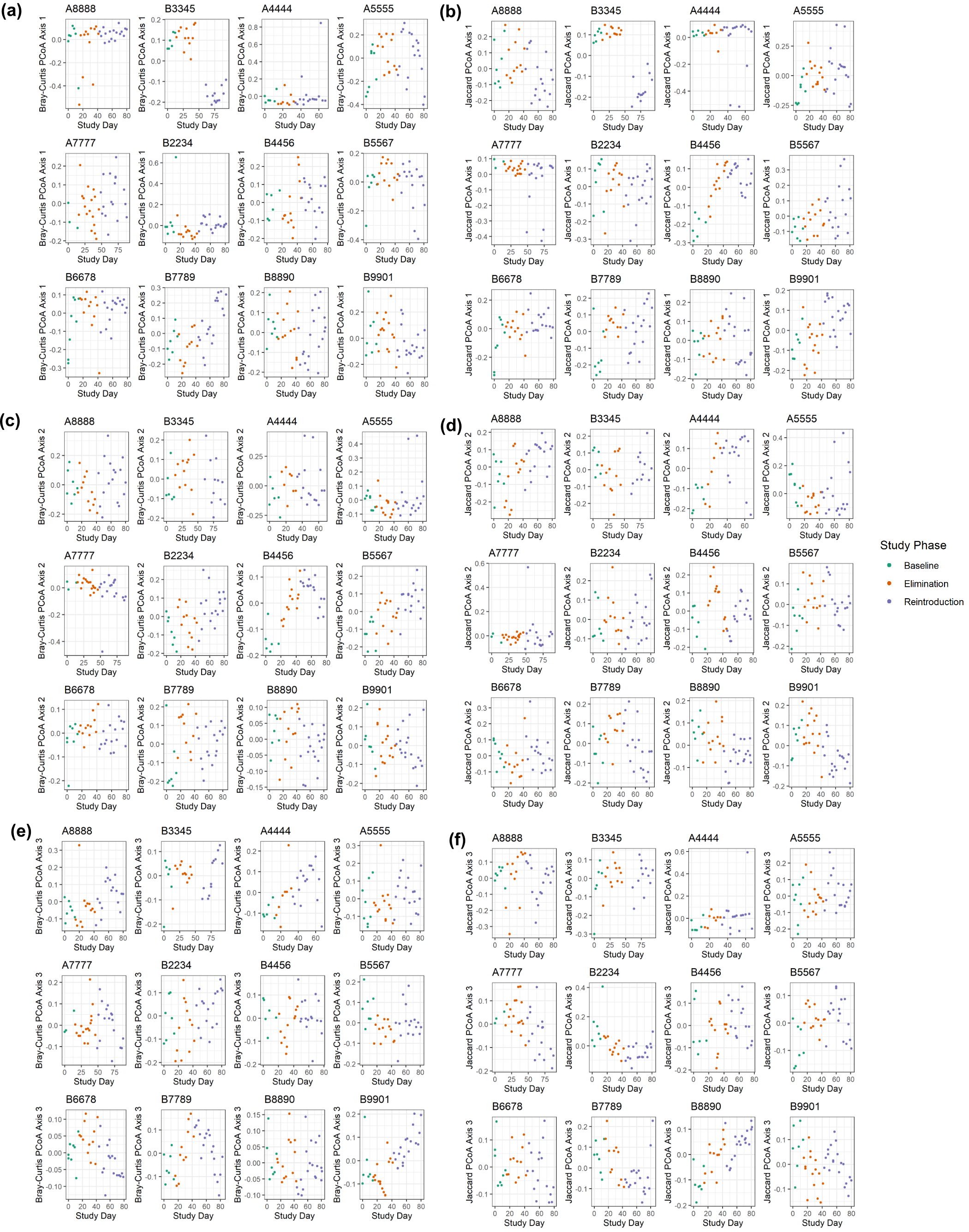

Supplement: FIG S8 [file mbio.01051-22-s0010.tif]
